# Cattle tick vaccine researchers join forces in CATVAC

**DOI:** 10.1186/s13071-016-1386-8

**Published:** 2016-02-24

**Authors:** Theo Schetters, Richard Bishop, Michael Crampton, Petr Kopáček, Alicja Lew-Tabor, Christine Maritz-Olivier, Robert Miller, Juan Mosqueda, Joaquín Patarroyo, Manuel Rodriguez-Valle, Glen A. Scoles, José de la Fuente

**Affiliations:** ProtActivity R&D, Cuijk, The Netherlands; ClinVet International, Bloemfontein, South-Africa; Tick Unit, International Livestock Research Institute ILRI, Nairobi, Kenya; Council for Scientific and Industrial Research (CSIR), Pretoria, Gauteng South-Africa; Institute of Parasitology, Biology Centre Czech Academy of Sciences, Ceske Budejovice, Czech Republic; The University of Queensland, Queensland Alliance for Agriculture & Food Innovation, St. Lucia, QLD Australia; Murdoch University, Centre for Comparative Genomics, Perth, WA Australia; The Genomics Research Institute, Department of Genetics, Faculty of Natural and Agricultural Sciences, University of Pretoria, Pretoria, South-Africa; Cattle Fever Tick Research Laboratory, Agricultural Research Service, United States Department of Agriculture, Edinburg, TX USA; Facultad de Ciencias Naturales, Universidad Autónoma de Querétaro, Queretaro, Queretaro Mexico; BIOAGRO/DVT Universidade Federal de Viçosa, Viçosa, MG Brazil; Animal Disease Research Unit, Agricultural Research Service, United States Department of Agriculture, Washington State University, Pullman, Washington USA; SaBio. Instituto de Investigación en Recursos Cinegéticos IREC CSIC-UCLM-JCCM, Ciudad Real, Spain; Department of Veterinary Pathobiology, Center for Veterinary Health Sciences, Oklahoma State University, Stillwater, OK USA

**Keywords:** CATVAC, Vaccine, Cattle, Tick, *Rhipicephalus microplus*

## Abstract

A meeting sponsored by the Bill & Melinda Gates Foundation was held at the Avanti Hotel, Mohammedia, Morocco, July 14–15, 2015. The meeting resulted in the formation of the Cattle Tick Vaccine Consortium (CATVAC).

## Control of cattle tick infestations: an urgent need in Africa

According to results from a continent-wide survey in Africa conducted in 2014–2015, ticks and tick-transmitted diseases, gastrointestinal helminth infections, and Peste des Petits Ruminants (PPR) are the most important diseases that affect the livelihood and development of communities in rural Africa that depend on livestock [1]. Extensive use of acaricides to control tick infestation has led to the selection for strains that are resistant against a number of these drugs [2]. This is particularly evident for the cattle tick *Rhipicephalus microplus* due to the fact that the different tick stages develop on a single host. Recently, it became apparent that this tick species is now spreading over even larger areas of the African continent, thus posing an immediate and further threat to livestock. Additional control methods are urgently required to limit tick populations [3].

Ever since it was shown that blood-feeding ectoparasites were affected when feeding on hosts that had been vaccinated with crude extracts of these arthropods, researchers aimed at developing effective vaccines [4, 5]. These efforts have met with some success in cattle tick vaccine research, but none of the vaccines appeared effective enough to sufficiently control tick infestation or transmission of the pathogens carried by them [5].

**Fig. 1:**
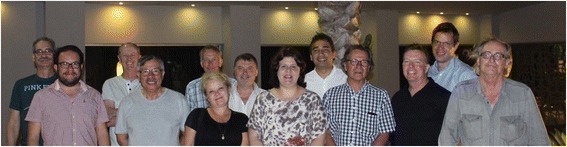
The participants of the official launch meeting of CATVAC. G.A. Scoles not on the picture. This meeting was held in Mohammedia, Morocco, July 14–15, 2015

Until now, there have been few attempts to study the effects of combinations of the partially protective tick antigens, which could potentially enhance vaccine efficacy against tick infestations and pathogen infection and transmission. In order to establish a closer collaboration between research groups that work on tick vaccines, a two-day meeting sponsored by the Bill & Melinda Gates Foundation was held on July 14-15^th^, 2015 at the Avanti Hotel in Mohammedia, Morocco. Key players in tick vaccine research from the different continents were invited to give an update on their research and to participate in the discussions. The purpose of the meeting was to exchange scientific knowledge, update the tick vaccine research, and select possible combinations of tick antigens that hold promise as an effective tick vaccine against cattle ticks, *R. microplus*. At the meeting it was also decided to establish a formal collaboration among the groups to facilitate exchange of antigens and evaluation of candidate vaccine formulations in standardized experimental models. This collaboration resulted in the formation of the Cattle Tick Vaccine Consortium (CATVAC; Fig. 1).

## Cattle tick vaccination studies

### Bm86/Bm95

The only recombinant tick vaccines on the market contain 100 μg of the immunoprotective midgut antigen Bm86 of *R. microplus* produced in *Pichia pastoris* and are formulated in water-in-oil adjuvants. On average, the level of protection obtained as judged by reduction of the number of engorged females after tick infestation is approximately 50 %, but this percentage depends on the tick species and strain involved [6]. Moreover, post-engorgement female mortality and effects on female fertility may reduce the number of infective larvae by up to a maximum of 90 %, depending on tick strains [6]. Additionally, Peter Willadsen indicated that Bm86 produced in *Escherichia coli* and baculovirus has also been successfully used as vaccine antigens [7].

### Full-length Bm86 antigen

In a comparative study performed in Texas, USA using Gavac® and a third party-derived formulation with Bm86, it was shown that efficacy against *R. microplus* was 27 and 50–65 % depending on the tick strain and adjuvant formulations used. However, vaccine efficacy was close to 100 % against *Rhipicephalus annulatus* strains [8]. These results corroborate the hypothesis that strain differences affect the vaccine efficacy against *R. microplus* that is observed in different models/experiments [9]. It was emphasized by a number of presenters that vaccination with Bm86 is generally more effective against *R. annulatus* than against *R. microplus* [10]. In addition, cattle vaccinated with Bm86 were significantly protected against infestation with *Rhipicephalus decoloratus*, but not against *Rhipicephalus appendiculatus* [11]. Cattle vaccination with the Bm86 homologue of *R. appendiculatus* affected moulting from nymphs to adults from 95.5 to 89.3 %, a statistically significant effect [12]. The antigen (approximately 100 μg protein per vaccine dose) was delivered subcutaneously as three separate inoculations at four week intervals in Montanide ISA 50 V (Seppic). Duration of immunity studies revealed that immunity induced with Bm86/Bm95 could last for 5–6 months, which has also been suggested after evaluation of the performance of the commercial vaccines TickGARD® and Gavac® over a period of 10 years in the field [6]. A single yearly booster vaccination is required to maintain this level of immunity. Gavac® is commercially available in Latin American countries such as Cuba and Venezuela. TickGARD® is not commercially available anymore but Alicja Lew-Tabor (The University of Queensland, Australia) mentioned that some stock is still available from Queensland University.

### Synthetic partial Bm86 antigen

Joaquín Patarroyo (Universidade Federal de Viçosa, Brazil) presented results in which a vaccine based on three peptides of the Bm86 protein that differ only at two amino acid positions from the original Australian tick sequence is being used in the field in Brazil with 2 mg of recombinant protein per dose with 1.5 mg of saponin as adjuvant [13]. Cattle were vaccinated three times with a 30-day interval. Protection against tick infestation is >80 % reduction in engorged females. This study, which includes a number of non-vaccinated control farms where acaricide treatment is used to control tick populations, is on its third year and involves 15,000 cattle head in total.

### Ferritin 2(Fer-2)

Fer-2 is a secreted molecule expressed in tick gut that functions in inter-tissue transport of non-heme iron originating from blood meal [14]. Vaccination of rabbits with Fer-2 from *Ixodes ricinus* expressed in *E. coli* provided protection against infestation with *I. ricinus* ticks (43 % reduction in numbers of engorged female ticks; [15]. The Fer-2 homologue of *R. microplus* adjuvanted with Montanide ISA 50 V also induced protection when used as a vaccine in one experiment using cattle and *R. microplus* tick infestation. The protective effect was reflected in the number of engorged females being reduced by 30 % and an additional effect on fertility resulting in a total efficacy of 64 % on viable progeny [15]. Petr Kopáček (Institute of Parasitology, Biology Centre Czech Academy of Sciences, Czech Republic) presented a repeat study showing only partial protection affecting mainly oviposition and larval hatching and additional repeat studies are ongoing. Kopáček also emphasized the vaccination potential of other molecules possibly playing a role in tick heme and iron metabolic pathways of *I. ricinus* that were recently screened using RNA-interference (RNAi) and experimental vaccination of rabbits. However, none of the tested molecules surpassed Fer-2 in their impact on the tick development and reproduction.

### Subolesin (SUB)

SUB is an intracellular regulatory protein that is involved in signal transduction. José de la Fuente (SaBio, Instituto de Investigación en Recursos Cinegéticos, Spain) reported that cattle vaccination with SUB from *R. microplus* adjuvated with Montanide ISA 50 V reduced the numbers of engorged female ticks after infestation with *R. microplus* by 47 % (60 % total efficacy considering the effect on oviposition and fertility; [5]). Similar results were found on vaccinated white-tailed deer. Additionally, partial protection has also been found for various hard and soft tick species and for other ectoparasites such as poultry red mite, mosquitoes and sand flies, hence its applicability in vaccines against haematophagous arthropods appears relatively wide [5]. Additionally, the effect of SUB vaccination on tick pathogen infection or transmission has been also documented, therefore suggesting its possible application to control vector infestations and pathogen infection [5]. A chimeric protein (Q38) that contains protective epitopes identified in SUB and the mosquito orthologue Akirin was used to vaccinate cattle (three intramuscular vaccinations; [16]). A single dose consisted of 100 μg protein produced in *E. coli* and formulated with Montanide ISA 50 V adjuvant. Upon infestation with *R. microplus*, a reduction of 69 % in the number of engorged females was observed. There was some additional effect on oviposition with 75 % total efficacy.

### SILK antigen

de la Fuente reported that the *R. microplus* SILK antigen was discovered for its role in tick-*Anaplasma marginale* interactions and used to vaccinate cattle with 3 intramuscular vaccinations [16]. A single dose consisted of 100 μg protein produced in *E. coli* and formulated with Montanide ISA 50 V adjuvant. Upon infestation with *R. microplus* a reduction of 58 % in the number of engorged females was observed. There was little additional effect on oviposition with 62 % total efficacy.

### New vaccine antigen candidates

A number of research groups are using reverse vaccinology or vaccinomics to discover new vaccine antigen candidates [5, 17]. In order to limit the number of putative vaccine candidates, a number of selection criteria is being used such as antigens that evoke antibodies that interfere with coagulation or in vitro feeding of adult ticks. Some of these candidates were presented that have been tested in vaccination-challenge experiments. These new candidates include (a) antigens interfering with blood coagulation and digestion (TCX, reprolysins), (b) antigens interfering with in vitro tick feeding (mix of 6 peptides), and (c) antigens interfering with tick biology (Aquaporins). A Kunitz-type protein with unknown function that is found on the cell surface of *R. microplus* midgut cells (TCX), was identified as one of three protein-binding partners of Bm86 using a yeast two-hybrid assay [18]. Upon infestation with *R. microplus* larvae, a reduction of 74 % in the number of engorged adult female ticks was found with a significant phenotype of “dandruff-like” nymphs and young adults. In studies using a combination of TCX and Bm86, the effect of Bm86 was increased four-fold. In addition, a combination of reprolysins, a group of metalloproteases from *R. microplus* [19] was used to vaccinate calves and induced approximately 60 % reduction in the number of engorged females. All antigens were produced in *E.coli* and calves were vaccinated with 100 μg antigen subcutaneously as three separate inoculations at four week intervals in Montanide ISA 50 V.

Lew-Tabor reported that a mix of 6 peptides that were selected because of the fact that antibodies against those peptides reduced the proportion of ticks that successfully completed a blood meal in vitro was used to vaccinate cattle [17]. Upon infestation with *R. microplus* larvae, a reduction of >85 % in the number of engorged adult female ticks was found. When each of these peptides was evaluated individually, protection was usually much less (15–45 %), but trials are on-going testing the use of new formulations with these putative protective antigens.

During feeding ticks concentrate the blood meal in the midgut by removing water and excreting it back into the host as saliva. Aquaporins are membrane proteins that have been identified as playing a major role in this process. Out of three aquaporin genes identified in *R. microplus*, the potential use of Aquaporin 1 (RmAQP1) as a vaccine was recently tested. In two independent pen trials conducted in Brazil using Holstein calves vaccinated with *Pichia*-expressed recombinant RmAQP1 there was a high efficacy of 75 and 68 %, respectively, mainly due to the clear reduction in number of adult ticks that fed successfully [20]. The importance of Aquaporin 2 (RmAQP2) was demonstrated by RNAi mediated blocking of expression of this gene. There was a significant reduction in tick reproductive fitness and larval viability [21]. Interestingly, Glen A. Scoles (USDA, Washington State University, USA) reported that this effect appeared to be higher when the ticks were infected with *Babesia bovis*.

### Combined antigens

In order to increase the level of protection by vaccination it has been suggested earlier to test combinations of antigens that each had shown to induce partial protection against tick infestations. A number of antigens have been used in combination with the Bm86 midgut protein with variable results. Theo Schetters (ProtActivity R&D, The Netherlands) reported on the use of Bm86 combined with SUB. Cattle that were vaccinated with 100 μg of Bm86 produced in *P. pastoris* and 50 μg of SUB produced in *E. coli* in separate vaccine formulations in Montanide ISA 50 V2 adjuvant were almost completely protected against *R. microplus* infestation (>95 % reduction in the number of engorged females; [22]). In vitro feeding studies using *R. microplus* larvae suggested that the effect is due to the synergistic activity of antibodies against each of the antigens. De la Fuente presented results using a chimeric protein composed of antigenic peptides from the Bm95 protein and the complete SUB protein fused to the *A. marginale* MSP1a protein to present the antigens on the surface of recombinant *E. coli* [23]. The bacterial membrane fraction containing 120 μg of recombinant chimeric protein per dose adjuvanted with Montanide ISA50V2 was given three times through the intramuscular route and induced 60 % reduction in *R. microplus* infestations and 91 % efficacy considering the reduction of viable progeny in a preliminary trial in cattle.

## Efficacy models

In order to evaluate the protective effects of cattle vaccination with tick antigens, a number of efficacy models are being used. Especially when using reverse vaccinology or vaccinomics, there is a need for efficient screening systems to select putative vaccine antigen candidates [5, 17]. Results obtained with different models were presented, and pros and cons were discussed.

### In vitro models

Blocking of gene expression using RNAi can be used to discover genes that are essential for tick biology, however, this strategy does not specifically select for vaccine antigen candidates. Because protective immunity against tick infestation is mainly antibody mediated, a system that shows the protective activity of serum from experimental animals that were vaccinated with tick vaccine formulations is needed. Preferably, antibodies should be raised in immunized cattle but smaller laboratory animals can also be used during this phase of antigen screening. Protective activity of serum raised against Bm86 was demonstrated using tube feeding of adult *R. microplus* adult ticks and membrane feeding of *R. microplus* larvae [22]. The latter system is expected to become publically available this year, and has the advantage over capillary tube feeding of semi-engorged adult females that it allows more efficient screening of vaccine candidate antigens. The application of this system for nymphs and adult ticks needs to be validated. Importantly, in vitro feeding systems allow combining sera with different antigen specificity to discover synergetic effects of antibodies raised against these individual antigens. This combination increases the efficiency of antigen screening, and reduces the use of experimental animals and development costs. It should be realized, however, that identification of effective combinations of protective antigens by these methods does not cover the entire spectrum of putative protective antigens as some antigens will be missed.

### In vivo models

Infestation of natural hosts with tick larvae is considered the best model to study the effects of vaccination on tick populations in the field. It was agreed at the meeting that vaccine efficacy should be expressed as reduction in the number of fully engorged adult ticks. There are essentially two techniques used: infestation at a confined space and whole body tick challenges. Confined tick infestations have been used for several tick species such as *R. appendiculatus* and *I. ricinus* using ear-wraps. However, as presented by Juan Mosqueda (Universidad Autónoma de Queretaro, Queretaro, Mexico), when studying ticks from the *Boophilus* group, nettings that are glued to the flank of the calf are usually applied, thus creating a patch where ticks feed. Confined infestations allow studying the effect of vaccination on different tick species on a single animal. This method has been used to study the effect of vaccination with Bm86 and SUB against *R. microplus* and *R. annulatus* [22]. It was generally agreed that whole body infestations reflect better a natural field infestation of cattle. For whole body infestations, larvae derived from tick colonies that are kept at the laboratory are put on the bovines at one or, preferably, more occasions at close intervals. In all cases, animals are restrained from grooming (e.g., with a short halter) for the duration of tick feeding to minimize grooming. Because ticks of the *Boophilus* group are one-host ticks, larvae will develop to nymphs and subsequently adults on the same animal. Fully engorged ticks that drop off from the animals in the third week after larval infestation must be recovered from the tray below the grid floor and purified from contaminating feces. Ticks can also be incubated to determine egg viability through the determination of percent emerging larvae. It should be clear that whole body tick infestations do not easily allow studying the effect of vaccination on different tick species, unless tick species determination using molecular biological techniques is additionally performed on ticks that are recovered from cattle. Schetters emphasized that when using whole body infestation, a minimal number of five cattle per experimental group is required to detect relevant statistical differences of at least 60 % reduction in the number of engorged female ticks.

Ultimately, field experiments are required to study the effect of vaccination on tick infestation. The major problem is that the natural level of infestation cannot be controlled, which increases the number of animals per experimental group including a control group to reach a sufficient statistical power. It is important to evaluate the different methodologies used in field studies and standardize these in order to make comparison between different field trials. There was some debate on whether a confined challenge that forces the ticks to feed on a part of the body that is not the preferred site for feeding, might not reflect the natural situation. It was argued that the fact that in the natural situation there are usually fewer ticks feeding on the flank than for instance on the neck of the animals is likely due to the fact that cattle groom themselves, and remove ticks from those areas. An experimental trial that compares the development of graded numbers of ticks applied at a patch or applied as a whole body infestation was suggested. An important parameter is the proportion of larvae that develops to fully engorged ticks. The consortium should agree upon a minimal value to consider a vaccine formulation as efficacious for the control of cattle tick infestations.

## The way forward

**Fig. 2:**
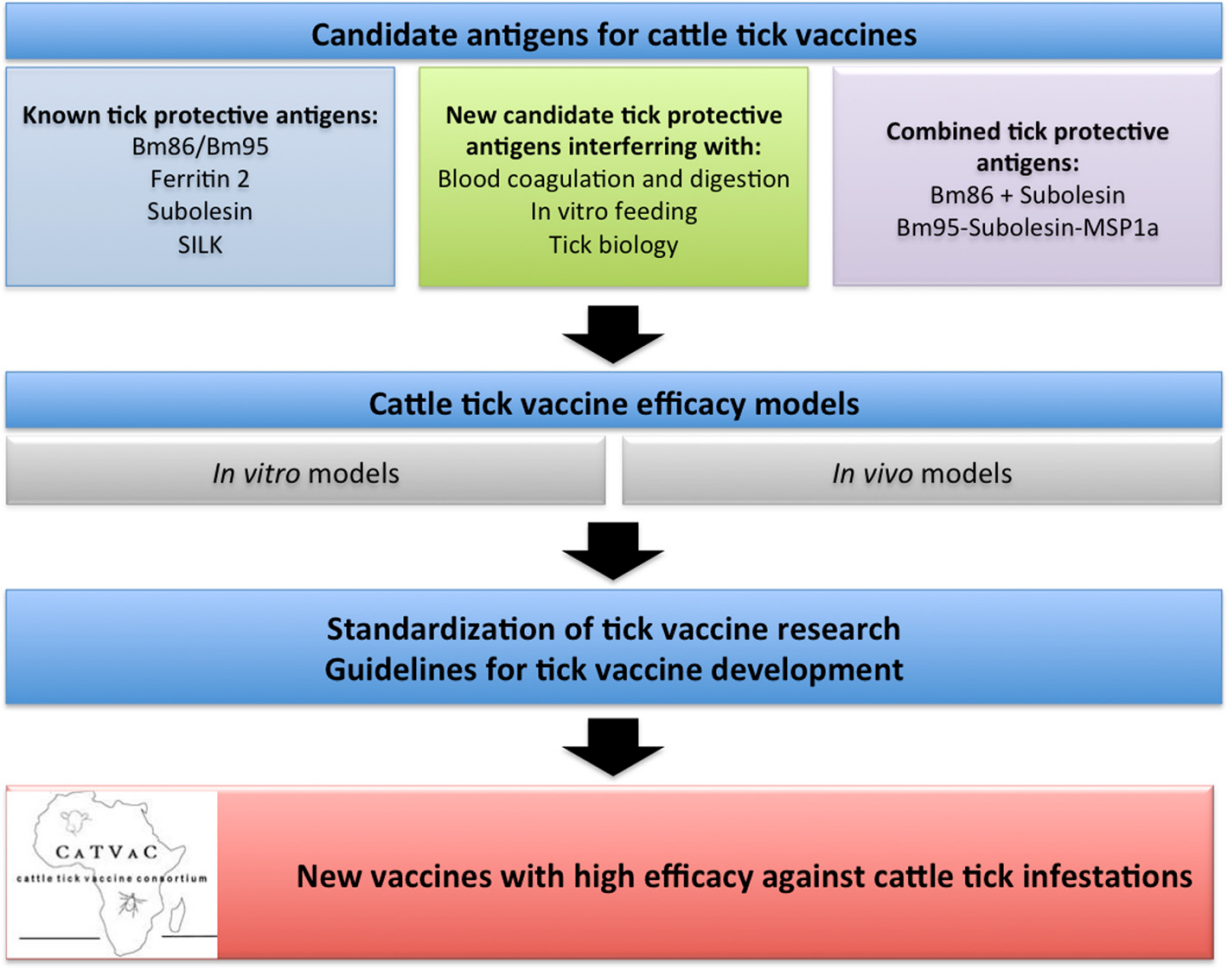
Working pipeline proposed by CATVAC for development of effective vaccines for cattle tick control

The meeting participants agreed on the pipeline for the development of effective vaccines for the control of cattle tick infestations (Fig. 2) and expressed their desire to continue this cattle tick vaccine initiative. It was suggested to formalize the formation of a Cattle Tick Vaccine Consortium (CATVAC). The consortium will be guided by a Steering Committee formed by Christine Maritz-Olivier, José de la Fuente and Theo Schetters (Chair) who will drive the project and take responsibility to realize the action points listed below.

### Standardization of tick vaccine research

Standardization of tick vaccine research is of ultimate importance to allow comparison of results obtained by different research groups. A number of specific standards were defined and included (a) positive antigen control, (b) challenge model, (c) calculation of efficacy, and (d) correlate of protection. The meeting agreed to establish a positive antigen control for vaccine efficacy studies based on Bm86. This control preparation will be used in future efficacy trials. In addition, a standardized antibody test to determine the antibody titres against Bm86 will be developed and shared among participants. The meeting also agreed to define specific parameters for in vivo challenge models. Parameters that can be standardized are the age of the experimental animals, sex, breed, tick strain/colony and number of larvae used for infestation, the minimal proportion of larvae that develop to fully engorged adults, and the challenge model (whole body or patch infestation). In order to improve clarity about efficacy of tick vaccines, especially the way this is communicated in abstracts of scientific publications that are published on line, it was decided to always present the percentage reduction in the number of engorged females upon infestation with *R. microplus* larvae. In the case that also the effect of vaccination on oviposition and/or larval viability has been determined, this will be expressed as total efficacy, clearly stating which parameters were used to calculate this value (see Box 2 in [5]). Research should aim at defining a correlate of protection, which is a requirement for registration of the vaccine. Preferably, the correlate of protection is the antigen content or the antibody titer against the vaccine antigen(s). Such correlate of protection will be used to establish a potency test for the release of vaccine batches after quality control.

### Guidelines for tick vaccine development

The standardization of tick vaccine research as described above forms the basis for the formulation of guidelines for tick vaccine development. These guidelines will be discussed with representatives of the World Association for the Advancement of Veterinary Parasitology (WAAVP) and submitted for publication in Veterinary Parasitology, the official organ of the WAAVP.
